# Supplementary figures and images for: Mapping responses to focal injections of bicuculline in the lateral parafacial region identifies core regions for maximal generation of active expiration
Source: eLife. 2024 Jul 17;13:RP94276. doi: 10.7554/eLife.94276 (PMC11254382; doi:10.7554/eLife.94276)

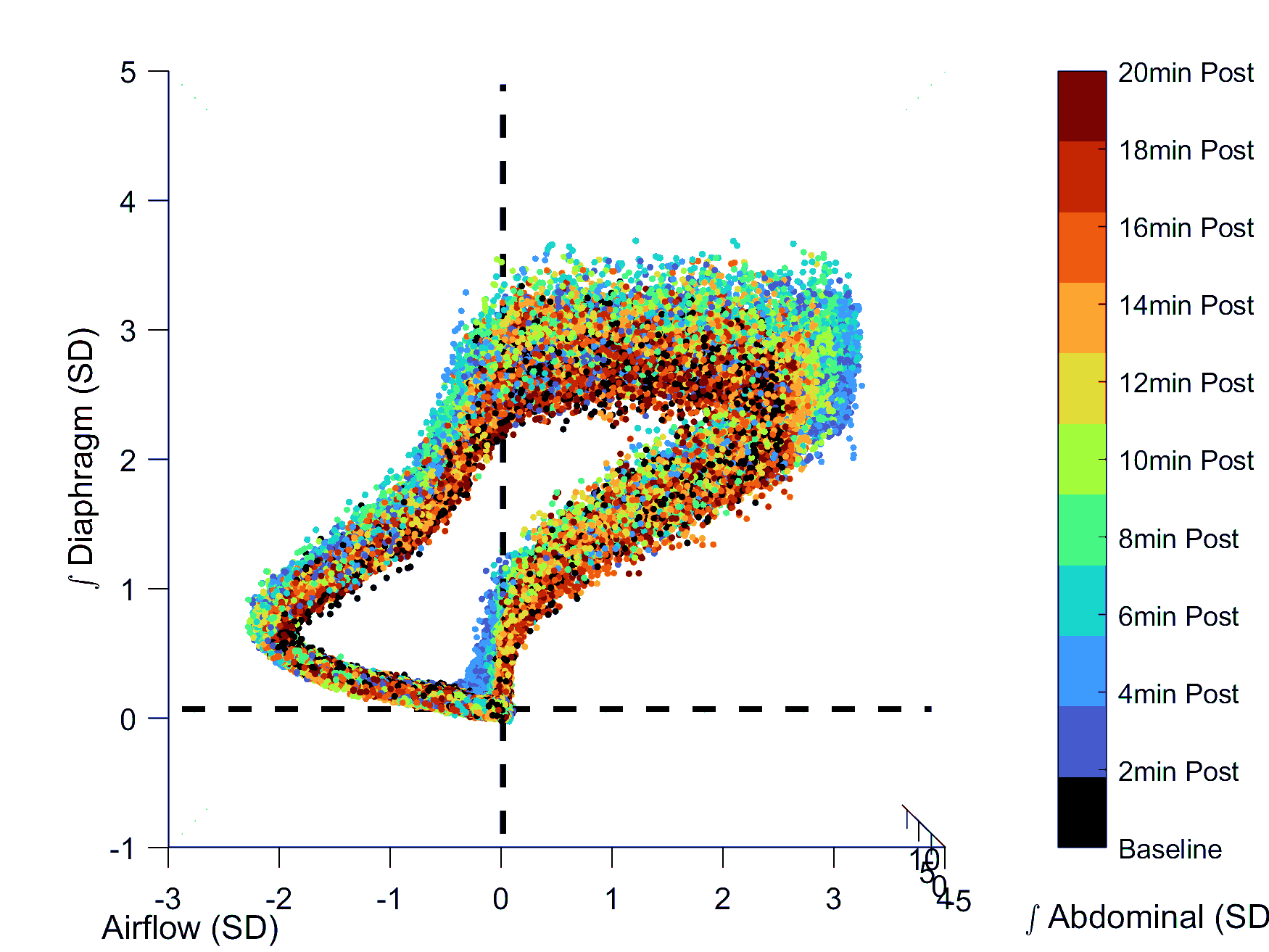

Supplement: Supplementary file 11 [file elife-94276-fig6-video1.gif]

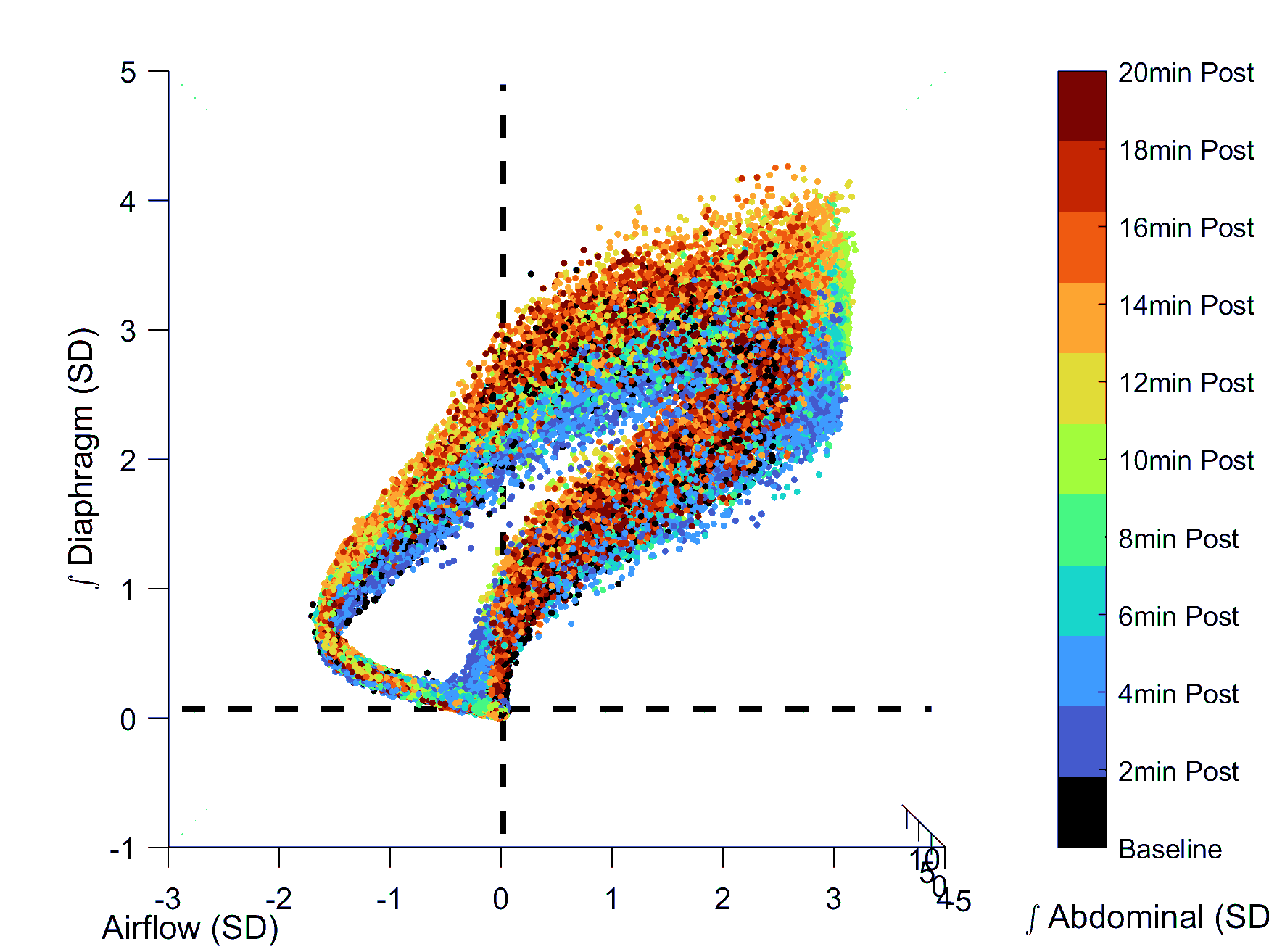

Supplement: Supplementary file 12 [file elife-94276-fig6-video2.gif]

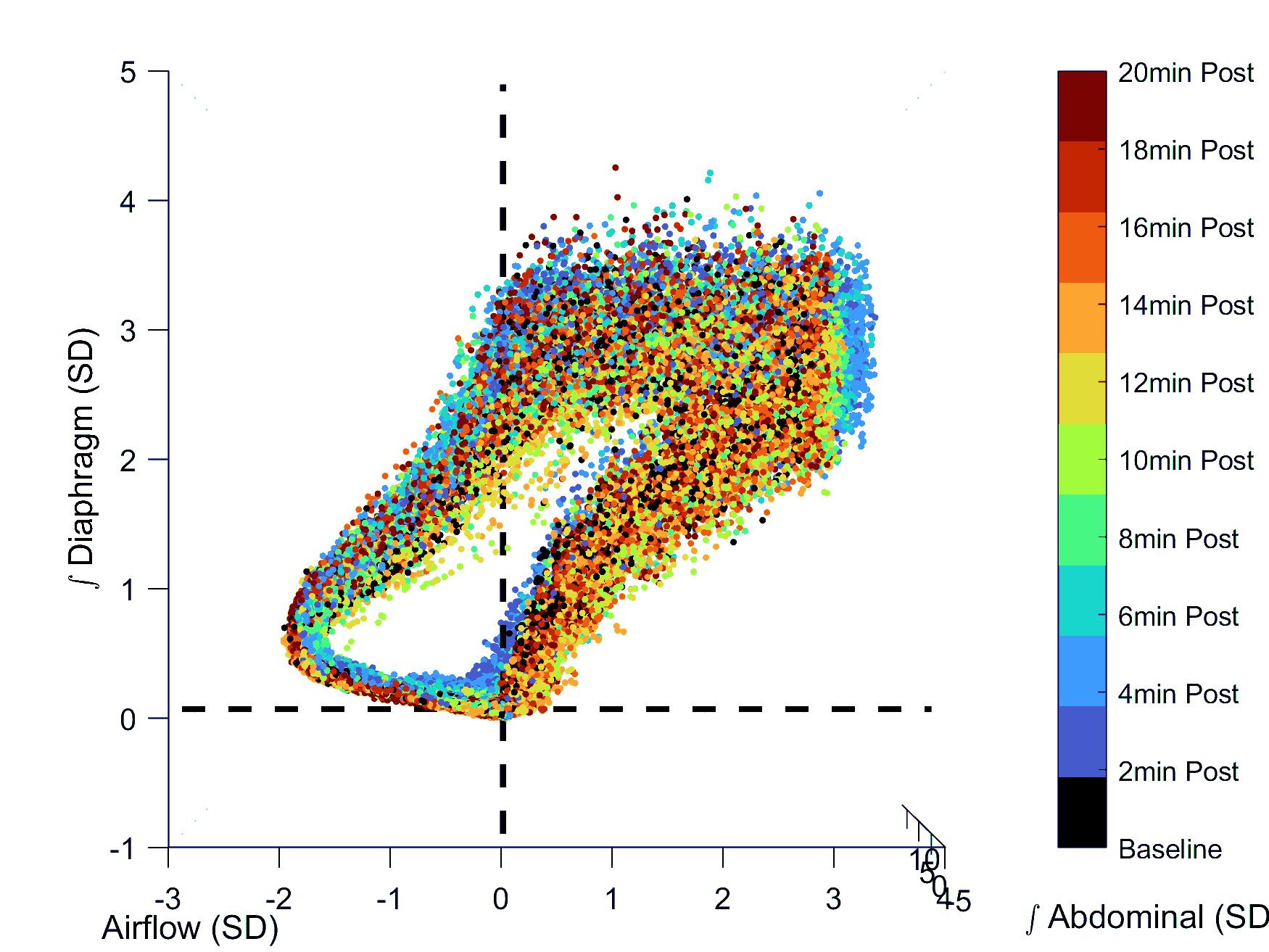

Supplement: Supplementary file 13 [file elife-94276-fig6-video3.gif]

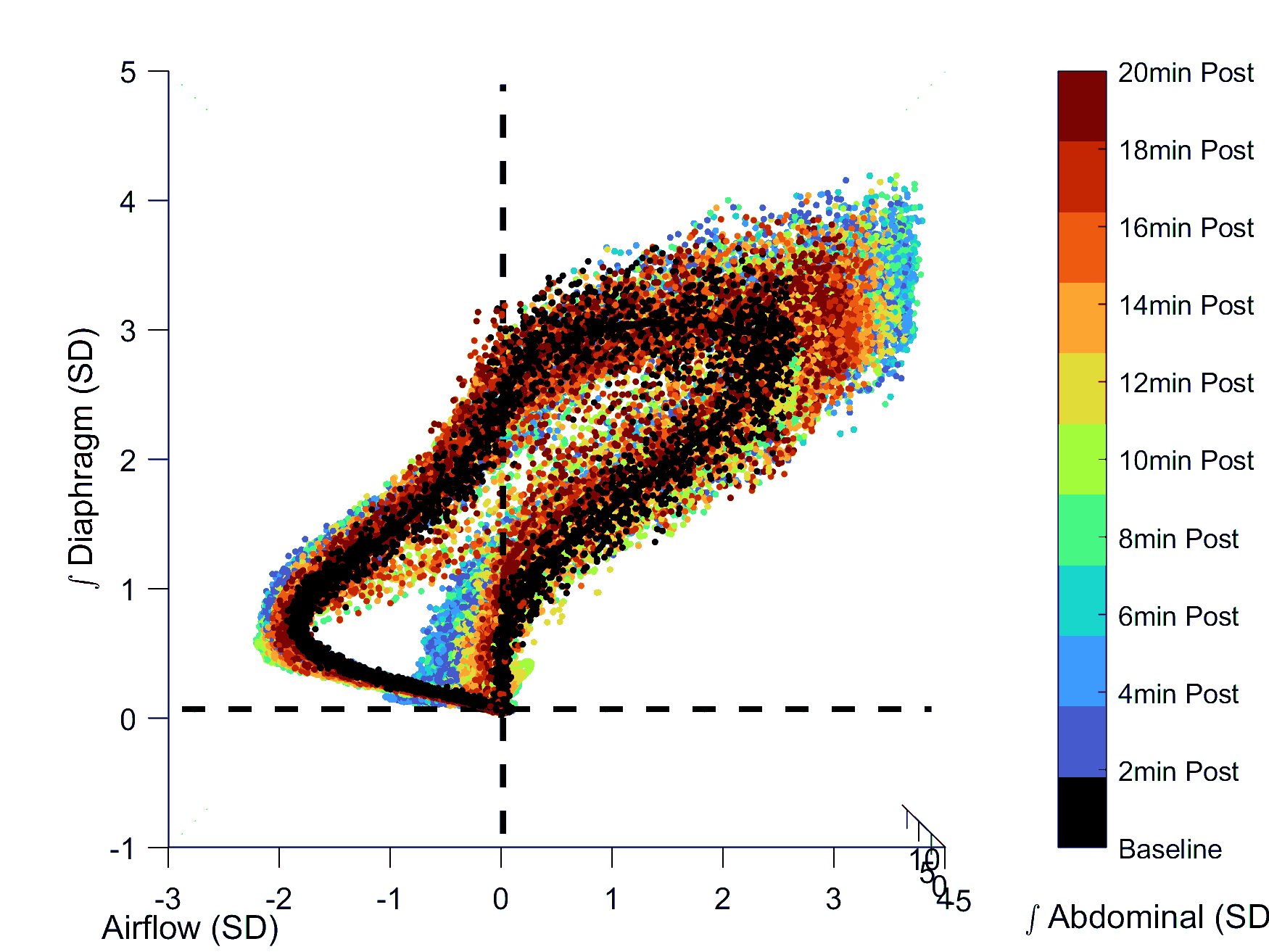

Supplement: Supplementary file 14 [file elife-94276-fig6-video4.gif]

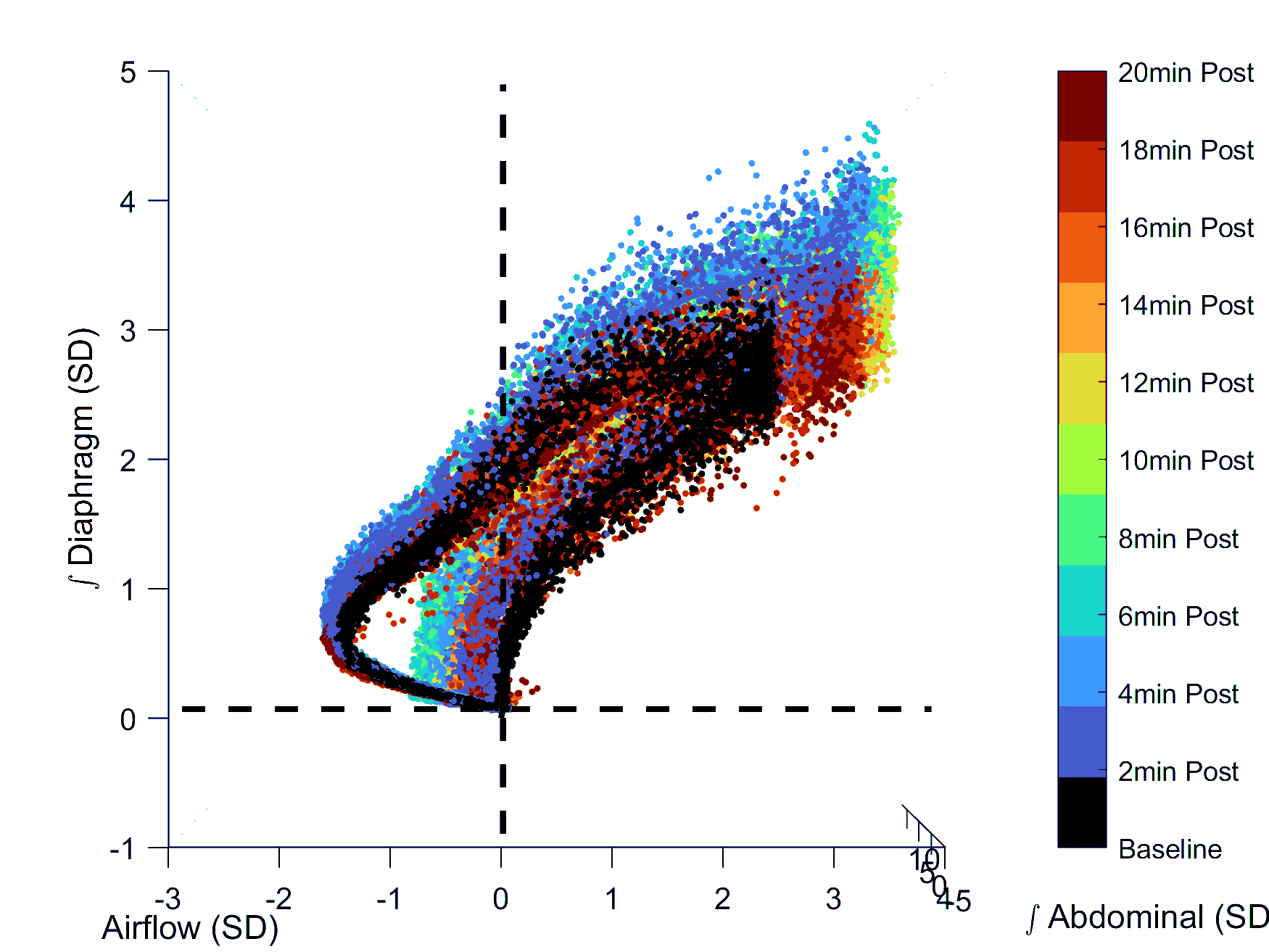

Supplement: Supplementary file 15 [file elife-94276-fig6-video5.gif]
